# Examining the overlap between tinnitus and depression questionnaires—protocol for an ICF based content analysis

**DOI:** 10.3389/fneur.2024.1376826

**Published:** 2024-09-03

**Authors:** Denise Fuchten, Adriana L. Smit, Inge Stegeman

**Affiliations:** ^1^Department of Otorhinolaryngology, Head and Neck Surgery, University Medical Center Utrecht, Utrecht, Netherlands; ^2^UMC Utrecht Brain Center, University Medical Center Utrecht, Utrecht, Netherlands

**Keywords:** tinnitus questionnaires, depression questionnaires, content analysis, overlap, International Classification of Functioning, Disability and Health (ICF)

## Abstract

**Introduction:**

Tinnitus is a common phenomenon with an estimated prevalence of 14.4% in the adult population. The experienced severity of tinnitus varies significantly among this population. Psychological factors have been identified as major contributors to this perceived severity, and numerous studies have demonstrated a correlation between symptoms of depression and tinnitus severity. However, the assessment of tinnitus severity and depressive symptoms often relies on self-report questionnaires, which show content overlap. This can pose challenges in distinguishing both conditions and interpreting their relationship. To address these challenges, the proposed study aims to examine the overlap between tinnitus and depressive symptom questionnaires by analyzing their content based on the International Classification of Functioning, Disability and Health (ICF) framework.

**Methods and analysis:**

Six validated, multi-item, self-report questionnaires measuring perceived tinnitus severity (THI, TQ, mTQ, THQ, TRQ, TFI) and seven validated, multi-item, self-report, depressive symptom questionnaires (BDI-II, HADS-D, SDS, PHQ-9, CES-D, SCL-90-R depression subscale, DASS-42 depression subscale) will be included in the content analysis. The content of all items of these questionnaires will be linked to ICF categories and item overlap between the tinnitus and depressive symptom questionnaires will be analyzed.

**Discussion:**

By exploring the overlap between depression and tinnitus questionnaires, this study seeks to gain a better understanding of the relationship between tinnitus and depression, by distinguishing between shared content and independent constructs of symptom scores and shedding light on the factors influencing their measured severity.

**Ethics and dissemination:**

Ethical approval is not required for this study, due to the characteristics of the study design. Findings will be disseminated through peer-reviewed open access publication and scientific conferences.

## 1 Introduction

Tinnitus, the perception of sound in the absence of an external auditory stimulus (1), is a common phenomenon with an estimated prevalence of 14.4% in the adult population, thereby affecting more than 740 million people worldwide (2). The experienced severity of tinnitus varies significantly among this population. While many people are unbothered by their tinnitus and can cope with it, others are severely impaired in their daily living (3). Recent estimates show that 2.3% of the adult population are bothered by their tinnitus or report that tinnitus influences their daily life, defined as `severe tinnitus’ in these estimates. This corresponds to 120 million people with `severe tinnitus’ globally (2).

Psychological factors have been identified as major contributors to this variation in perceived severity (4, 5), and numerous studies have demonstrated a correlation between (symptoms of) depression and tinnitus severity (6–9). Studies examining this relationship often utilize self-report questionnaires (10), such as the Tinnitus Handicap Inventory (THI) for assessing tinnitus severity and the Beck Depression Inventory (BDI) to evaluate symptoms of depression (9). However, these questionnaires show content overlap, which can pose challenges in accurately distinguishing both conditions, interpreting their relationship and applying the right treatment to reduce symptoms. For instance, Ooms et al. (5) identified content overlap in 15 out of 25 questions of the THI with 13 out of 21 questions of the BDI, and proposed this could potentially lead to an overestimation of the correlation between depression and tinnitus severity.

The content overlap observed in such questionnaires can partially be attributed to shared symptoms among tinnitus and depression. Patients with tinnitus often report complaints such as problems with sleeping, problems with concentration, social withdrawal and despair – symptoms that are also indicative of depression (10). Furthermore, variations exist in the content included among different tinnitus and depressive symptom questionnaires. Some tinnitus questionnaires for instance focus more on psychological distress, while certain depressive symptom questionnaires exclude somatic symptoms, which may also explain some of the variance when examining the relation between tinnitus and depression (9).

To further examine and understand the relationship between tinnitus and depression beyond potential method artifacts caused by the content overlap of the questionnaires, it is imperative to critically examine the methodology for measuring these constructs. In this manuscript, we therefore present a protocol for a study in which we aim to examine the overlap between tinnitus and depressive symptom questionnaires by analyzing their content based on the International Classification of Functioning, Disability and Health (ICF) framework. By adopting this approach, we seek to gain a better understanding of the relationship between tinnitus and depression, by distinguishing between shared content and independent constructs of symptom scores and shedding light on the factors influencing their measured severity.

## 2 Methods and analysis

### 2.1 Tinnitus questionnaires

The content analysis will include a selection of validated, multi-item, self-report questionnaires for assessing perceived tinnitus severity. These questionnaires have been widely utilized in both clinical practice and research in order to assess the severity of symptoms, and are recommended or referenced in multiple international clinical practice guidelines (11–18). Table 1 provides a summary of the characteristics of the included questionnaires.

**Table 1:** Characteristics of the tinnitus questionnaires.

Instrument	Subscales/factors	Items	Response options	Score range/grading	Cronbach’s α
THI (19)	Functional Emotional Catastrophic	11 9 5	4 Yes 2 Sometimes 0 No	0-100; higher total or subscale score indicates higher levels of tinnitus severity. 0-16 Slight 18-36 Mild 38-56 Moderate 58-76 Severe 78-100 Catastrophic (20)	0.93 (19)
		25			
TQ (21, 22)	Emotional distress Auditory perceptual difficulties Intrusiveness Sleep disturbance Somatic complaints Remaining items	19 7 7 4 4 11	2 True 1 Partly true 0 Not true ^a^	0-82^b^; higher total or subscale score indicates higher levels of tinnitus severity.	0.95 (22)
		52			
TQ – German version (23)	Emotional distress Cognitive distress Auditory perceptual difficulties Intrusiveness Sleep disturbance Somatic complaints Remaining items	12 8 7 8 4 3 12	2 True 1 Partly true 0 Not true ^a^	0-84^b^; higher total or subscale score indicates higher levels of tinnitus severity. 0-30 Mild 31-46 Moderate 47-59 Severe 60-84 Very severe	0.93 (24)
		52^c^			
mTQ (25)	None	12	2 True 1 Partly true 0 Not true	0-24; higher total score indicates higher levels of tinnitus severity. 0-7 Compensated 8-12 Moderately distressed 13-18 Severely distressed 19-24 Most severely distressed	0.87-0.90 (25)
THQ (26)	Physical, emotional and social consequences Hearing difficulty Personal viewpoint	15 8 4	0-100 Strongly disagree - Strongly agree	0-100; higher total or subscale score indicates higher levels of tinnitus severity.	0.94 (26)
		27			
TRQ (27)	General distress Interference Severity Avoidance	15 9 8 3	4 Almost all of the time 3 A good deal of the time 2 Some of the time 1 A little of the time 0 Not at all	0-104; higher total score indicates higher levels of tinnitus severity.	0.96 (27)
		26^c^			
TFI (29)	Intrusive Sense of control Cognitive Sleep Auditory Relaxation Quality of life Emotional	3 3 3 3 3 3 4 3	0-10 ^d^ Anchors vary between items	0-100; higher total or subscale score indicates higher levels of tinnitus severity. 0-17 Not a problem 18-31 Small problem 32-53 Moderate problem 54-72 Big problem 73-100 Very big problem (30)	0.97 (29)
		25			

*THI, Tinnitus Handicap Inventory; TQ, Tinnitus Questionnaire; mTQ, mini Tinnitus Questionnaire; THQ, Tinnitus Handicap Questionnaire; TRQ, Tinnitus Reaction Questionnaire; TFI, Tinnitus Functional Index.*

Notes: ^a^ Some items are scored reversed, ^b^ Only items included in the subscales are used to determine the total score, ^c^ Items can belong to multiple subscales/factors, ^d^ Two items need to be transformed from 0-100 to 0-10.

#### 2.1.1 Tinnitus Handicap Inventory

The Tinnitus Handicap Inventory (THI), developed by Newman et al. (19), serves as a self-report tinnitus handicap measure to quantify the impact of tinnitus on daily living. It is comprised of 25 items that can be divided into three subscales; functional, emotional and catastrophic. The functional scale addresses impairments in mental, social and physical functioning, while the emotional subscale assesses affective responses to tinnitus. The catastrophic subscale examines the catastrophizing reactions to tinnitus, such as loss of control, feeling unable to cope with tinnitus and feeling like you have a terrible disease. Each item of the questionnaire has three response options; yes (4), sometimes (2) and no (0). The total score on the THI ranges from 0 to 100, with a higher score indicating a higher level of tinnitus severity. Additionally, the THI provides a grading system that classifies scores from slight to catastrophic (20).

#### 2.1.2 Tinnitus Questionnaire

The Tinnitus Questionnaire (TQ), developed by Hallam et al. (21), assesses the psychological effects of tinnitus and is originally designed to evaluate cognitive therapy outcomes (22). The instrument consists of 52 items, covering five domains: emotional distress, intrusiveness, auditory perceptual difficulties, sleep disturbance and somatic complaints. Each item has three response options; not true (0), partly true (1) and true (2). A few items are scored reversed and therefore need to be converted to calculate the total score. The total score ranges from 0 to 82, incorporating only the 41 items included in the five subscales. Higher scores on the TQ indicate higher levels of psychological distress caused by tinnitus.

A German version of this questionnaire was developed by Hiller and Goebel (23), and scoring of the TQ often follows the criteria established in this version, as more data is available about its psychometric qualities. The German scoring method attributes items to six subscales as opposed to the original five subscales: emotional distress, cognitive distress, intrusiveness, auditory perceptual difficulties, sleep disturbance and somatic complaints. The total score ranges from 0-84, based on the 40 items included in the subscales, with two items scored twice. A higher score indicates greater tinnitus distress.

#### 2.1.3 Mini Tinnitus Questionnaire

The mini Tinnitus Questionnaire (mTQ), developed by Hiller and Goebel (25), is an abridged version of the TQ. It is created with the aim to be used as a quick tool for the assessment of psychological distress caused by tinnitus. The mTQ consists of 12 items selected from the TQ, each with three response options; not true (0), partly true (1) and true (2). The questionnaire only provides a total score, it is not possible to calculate subscale scores. A higher total score indicates higher levels of distress, with scores ranging from 0 to 24. A grading system is suggested, classifying scores from compensated to most severely distressed.

#### 2.1.4 Tinnitus Handicap Questionnaire

The Tinnitus Handicap Questionnaire (THQ), developed by Kuk et al. (26), measures the perceived degree of handicap caused by tinnitus. It consists of 27 items for which individuals indicate their degree of agreement on a scale from 0 (strongly disagree) to 100 (strongly agree). The questionnaire consists of three subscales; (1) physical, emotional, and social consequences of tinnitus, (2) hearing ability of the patient and (3) patients’ view of tinnitus. Due to the low internal consistency of the third subscale, it is recommended to only score the first two subscales. Both the subscale scores and the total score range from 0 to 100, with a higher score indicating greater tinnitus severity.

#### 2.1.5 Tinnitus Reaction Questionnaire

The Tinnitus Reaction Questionnaire (TRQ), developed by Wilson et al. (27), is designed to assess the psychological distress associated with tinnitus. The questionnaire consists of 26 items asking about tinnitus complaints over the past week, and although it utilizes no subscales, factor analysis revealed a structure with four factors; general distress, interference with work and leisure activities, severe signs of distress, and avoidance of activities. Items can be scored on a five-point scale ranging from 0 (not at all) to 4 (almost all of the time). The total score on the TRQ ranges from 0 to 104, with a higher score indicating higher levels of tinnitus related psychological distress.

#### 2.1.6 Tinnitus Functional Index

The Tinnitus Functional Index (TFI), developed by Meikle et al. (28), measures the severity and negative impact of tinnitus. It is designed to be able to evaluate treatment-related changes in tinnitus and to provide coverage of multiple domains of tinnitus severity. The 25 items of the questionnaire are divided into eight subscales; intrusive, sense of control, cognitive, sleep, auditory, relaxation, quality of life and emotional. Items can be scored on a scale from 0 to 10, with anchors varying between the items. Both subscale scores and a total score can be calculated, ranging from 0 to 100, with a higher score indicating higher levels of tinnitus severity. A grading system, classifying scores from not a problem to a very big problem, is available (30).

### 2.2 Depressive symptom questionnaires

For this content analysis, we aimed to include depressive symptom questionnaires that are commonly used in research on tinnitus. Based on two systematic reviews on tinnitus and depression (8, 9), seven validated, multi-item, self-report questionnaires are included. It is important to note that these questionnaires alone cannot provide a diagnosis of depression, and merely serve to assess the presence of symptomatology. They can therefore be used in general healthcare settings and research. Table 2 provides a summary of the characteristics of the included questionnaires.

**Table 2:** Characteristics of the depressive symptom questionnaires.

Instrument	Subscales/factors	Items	Response options	Score range/grading	Crobach’s α
BDI-II (31)	Cognitive Affective Somatic	7 5 9	0-3 Anchors vary between items	0-63; higher total score indicates more (severe) depressive symptoms. 0-13 Minimal depression 14-19 Mild depression 20-28 Moderate depression 29-63 Severe depression	0.93 (31)
		21			
HADS (depression subscale) (32)	N/A^a^	7/14	0-3 Anchors vary between items	0-21; higher total score indicates more (severe) depressive symptoms. 0-7 Normal 8-10 Mild 11-14 Moderate 15-21 Severe (34)	0.82 (33)
SDS (35)	None	20	1 A little of the time 2 Some of the time 3 Good part of the time 4 Most of the time ^b^	25-100; higher total score indicates more (severe) depressive symptoms. 25 – 49 Normal range 50-59 Mildly depressed 60-69 Moderately depressed ≥70 Severely depressed	0.86 (36)
PHQ-9 (44)	None	9	0 Not at all 1 Several days 2 More than half the days 3 Nearly every day	0-27; higher total score indicates more (severe) depressive symptoms. 1-4 Minimal depression 5-9 Mild depression 10-14 Moderate depression 15-19 Moderately severe depression 20-27 Severe depression Or; Suspected major depressive disorder: ≥5 items scored ≥2 (except for the suicidal ideation item, which counts if it is ≥1), and 1 of the symptoms = depressed mood or anhedonia. Suspected other depressive disorder: 2-4 items scored ≥2 (except for the suicidal ideation item, which counts if it is ≥1), and 1 of the symptoms = depressed mood or anhedonia (37).	0.86-0.89 (38)
CES-D (39)	Depressed affect Positive affect Somatic and retarded activity Interpersonal Remaining items	5 4 7 2 2	0 Rarely or none of the time (less than 1 day) 1 Some or a little of the time (1-2 days) 2 Occasionally or a moderate amount of the time (3-4 days) 3 Most or all of the time (5-7 days)	0-60; higher total score indicates more (severe) depressive symptoms. >16 indicator for the presence of depression	0.85-0.90 (39)
		20			
SCL-90-R (depression subscale) (40)	N/A^a^	13/90	0 Not at all 1 A little bit 2 Moderately 3 Quite a bit 4 Extremely	0-52; higher total score indicates more (severe) depressive symptoms.	0.89 (41)
DASS-42 (depression subscale) (42)	N/A^a^	14/42	0 Did not apply to me at all 1 Applied to me to some degree, or some of the time 2 Applied to me to a considerable degree, or a good part of the time 3 Applied to me very much, or most of the time	0-42; higher subscale score indicates more (severe) depressive symptoms. 0-9 Normal 10-13 Mild 14-20 Moderate 21-27 Severe ≥ 28 Extremely severe	0.96 (43)

*BDI-II, Beck Depression Inventory-II; HADS, Hospital Anxiety and Depression Scale; SDS, Zung Self-Rating Depression Scale; PHQ-9, Patient Health Questionnaire-9; CES-D; Center for Epidemiologic Studies Depression Scale; SCL-90-R, Symptom Checklist-90-Revised; DASS-42, Depression Anxiety Stress Scale 42.*

Notes: ^a^ This instrument is a subscale of a questionnaire, ^b^ Some items are scored reversed.

#### 2.2.1 Beck Depression Inventory-II

The Beck Depression Inventory-II (BDI-II), developed by Beck et al. (31), is a widely used self-report inventory measuring the severity of depressive symptoms. The questionnaire is a revised version of the BDI-I and is based on the criteria for depressive disorder of the Diagnostic and Statistical Manual of Mental Disorders 4th edition (DSM-IV). It consists of 21 items, asking about the way people have been feeling during the past 2 weeks. These items can be categorized in three subscales; cognitive, affective and somatic symptoms. Each item can be scored on a scale from 0–3. The total score of this inventory ranges from 0 to 63, with higher scores indicating more severe depressive symptoms. Additionally, a grading system is available to classify scores from minimal to severe levels of depression.

#### 2.2.2 Hospital Anxiety and Depression Scale—depression subscale

The Hospital Anxiety and Depression Scale (HADS), developed by Sigmond and Snaith (32), measures anxiety and depression, without including items on somatic complaints. The scale is divided into an anxiety and a depression subscale, both containing seven items asking about how people have been feeling during the past week. In the current content analysis only the depression subscale, called the HADS-D, will be analyzed. Each item consists of four response options, which can be scored on a scale from 0 to 3. The total score on this subscale ranges from 0 to 21, with higher scores indicating more depressive symptoms. A grading system, classifying scores from normal to severe, is available for the subscale (34).

#### 2.2.3 Zung Self-Rating Depression Scale

The Zung Self-Rating Depression Scale (SDS), developed by Zung (35), measures the severity of depressive symptoms. It is designed by including commonly found characteristics of depression; pervasive effects, physiological equivalents, psychomotor activities and other disturbances. It however does not contain subscales. The questionnaire consists of 20 items on how people felt or behaved during the past several days, each with four response options ranging from a little of the time to most of the time, that can be scored on a scale from 1 to 4. Half of the items are scored reversed and therefore need to be converted to calculate the total score. The total score on this scale ranges from 25 to 100, with higher scores indicating more severe depressive symptoms. Additionally, scores can be classified from normal range to severely depressed, using the available grading system.

#### 2.2.4 Patient Health Questionnaire-9

The Patient Health Questionnaire-9 (PHQ-9), developed by Kroenke et al. (44), is the nine-item depression module of the PHQ. The nine items, asking about symptoms over the past 2 weeks, are based on the DSM-IV criteria for depressive disorder. At the end of the questionnaire, an extra 10th item is added, asking patients about the impact of these complaints on their daily life. Items can be scored on a four-point scale ranging from 0 (not at all) to 3 (nearly every day). There are different methods for scoring the PHQ-9 (37). With the method based on the DSM-IV criteria, a major depressive disorder is suspected if five or more of the nine depressive symptom criteria are present for more than half of the days (except for the suicidal ideation item (item 9), which counts if it is scored at all), and one of the symptoms needs to be depressed mood or anhedonia. Other depressive disorder is suspected if 2–4 of the symptoms are present more than half of the days and one of the symptoms is depressed mood or anhedonia. The PHQ-9 can also be scored by adding up the scores of the items, with a total score range of 0–27. Higher scores indicate more (severe) depressive symptoms, and a grading system is available classifying the summed scores from minimal to severe depression.

#### 2.2.5 Center for Epidemiologic Studies Depression Scale

The Center for Epidemiologic Studies Depression Scale (CES-D), developed by Radloff (39), is a structured self-report scale developed for the use in studies on the epidemiology of depressive symptomatology in the general population. The scale consists of 20 items asking about symptoms during the past week, with factor analysis showing four underlying factors: depressed affect, positive affect, somatic and related activity, and interpersonal. These factors however, are not considered as subscales in the questionnaire. Items can be scored on a four-point scale ranging from 0 (rarely or none of the time) to 3 (most or all of the time). The total score on the scale ranges from 0 to 60, with higher scores indicating the presence of more symptomatology. A score higher than 16 is indicative for the presence of a depression.

#### 2.2.6 Symptom Checklist 90-Revised—depression subscale

The Symptom Checklist-90-Revised (SCL-90-R), developed by Derogatis (40), is a 90-item multidimensional instrument for the assessment of psychological symptoms and psychological distress. The items ask about symptoms during the past week and are assigned to nine dimensions; somatization, obsessive-compulsive, interpersonal sensitivity, depression, anxiety, hostility, phobic anxiety, paranoid ideation and psychoticism. In the current content analysis only the depression domain will be analyzed. This domain consists of 13 items, with response options ranging from not at all (0) to extremely (4). The total score of the subscale ranges from 0 to 52, with higher scores indicating more severe depressive symptoms.

#### 2.2.7 Depression Anxiety Stress Scale 42—depression subscale

The Depression Anxiety Stress Scale 42 (DASS-42), developed by Lovibond and Lovibond (42), is a 42-item instrument measuring the negative emotional states of depression, anxiety and stress. In the content analysis only the depression subscale will be analyzed. This subscale consists of 14 items referring to symptoms over the past week, which can be scored from 0 (did not apply to me at all) to 3 (applied to me very much, most of the time). The total subscale score ranges from 0 to 42, with higher scores indicating more severe depressive symptoms, and a grading system is available classifying the score from normal to extremely severe.

### 2.3 ICF Linking Procedure

The International Classification of Functioning, Disability and Health (ICF), is a framework developed by the World Health Organization (WHO) to serve as an international standard to describe and measure health and disability (45). The ICF framework contains hierarchically organized categories that are divided into two parts. Part 1 ‘functioning and disability’ covers body structures, body functions, and activities and participation. Part 2 ‘contextual factors’ covers environmental factors and personal factors. All components, except for the component personal factors, contain chapters (first-level categories), that are further divided into second-, third- and sometimes fourth-level categories with a corresponding code. Table 3 illustrates an example of the hierarchical structure of the ICF framework.

**Table 3:** Example of the hierarchical structure of the ICF Framework.

Level	Example	Coding
Component	Body Functions	b
Chapter (first level)	Sensory functions and pain	b2
Second level	Sensations associated with hearing and vestibular function	b240
Third level	Ringing in ears or tinnitus	b2400

The content analysis of the tinnitus and depressive symptoms questionnaires will be performed by linking each questionnaire item to an ICF category based on the linking rules developed and refined by Cieza et al. (46–48). These linking rules provide a standardized and systematic approach to connect information to the ICF framework. In accordance with these rules, the main concept and additional concepts of each item of the questionnaires will be identified, after which they will be linked to the most precise ICF category. An item can be assigned to more than one category when the item contains multiple concepts. If a concept is not covered in the ICF framework, the code ‘nc’ (not covered) will be assigned. The code ‘pf’ will be assigned if the concept of an item is considered to be a personal factor, and is therefore not further categorized in the ICF framework. When the information provided by a questionnaire item is not sufficient for linking it to an ICF category, the code ‘nd’ (not definable) will be assigned. Further elaboration on the most recent version of the linking rules can be found in the article of Cieza et al. (48). All items will be linked by two independent reviewers. A third reviewer will be consulted if needed for reaching consensus.

### 2.4 Data analysis

Content analysis will be performed on the second level ICF categories assigned to each item of the included questionnaires. Frequencies of the assigned categories in each questionnaire will be reported as absolute numbers and percentages. Each tinnitus questionnaire will be compared to each depressive symptom questionnaire to define the amount of item overlap between questionnaires; the number of items that are linked to the same category in the two compared questionnaires. Overlap will be reported as absolute numbers and percentages, and a cross table will be made to visualize the amount of content overlap between the questionnaires. Cohen’s Kappa coefficient will be reported for the inter-rater reliability between the two screeners on the second level categorization.

## 3 Discussion

Distinguishing tinnitus and depression symptoms and interpreting their relationship can be a challenging task considering the overlap in self-report questionnaires that are commonly used in assessing the symptoms and severity of these constructs (5, 9, 10). With the proposed study we therefore aim to examine the overlap between tinnitus and depressive symptom questionnaires by analyzing their content based on the ICF framework.

By using this standardized and internationally recognized framework to guide the content analysis, this study ensures a systematic examination of the items in the questionnaires. This will facilitate the comparison of the content across different measures and improves the comprehension of the results of these measures. Furthermore, multiple validated and widely established questionnaires assessing tinnitus severity and symptoms of depression will be included, which will not only provide a comprehensive understanding of the similarities and differences between the measures, but also a greater understanding of the constructs underlying tinnitus and depression severity scores. Examining the overlap will contribute to our ability to interpret the relationship between tinnitus and depression, and might help us in applying the right intervention to reduce symptoms. It is however important to acknowledge that individual patient interpretations of questionnaire items might differ from the determined content. While the content analysis based on the ICF framework offers a structured and systematic approach, it may not capture the full range of patient experiences and perspectives. Future research could consider incorporating methods to analyze overlap based on the patient perspective, to gain an even further understanding of content overlap.

## Ethics statement

Ethical approval is not required for this study, due to the characteristics of the study design. Findings will be disseminated through peer-reviewed open access publication and scientific conferences.

## Author contributions

DF: Methodology, Writing – review & editing, Writing – original draft, Conceptualization. AS: Methodology, Writing – review & editing, Writing – original draft, Conceptualization. IS: Methodology, Writing – review & editing, Writing – original draft, Conceptualization.
